# Salivary gland stem/progenitor cells: advancing from basic science to clinical applications

**DOI:** 10.1186/s13619-025-00221-5

**Published:** 2025-01-24

**Authors:** Jimpi Langthasa, Li Guan, Shyam Lal Jinagal, Quynh-Thu Le

**Affiliations:** 1 Department of Radiation Oncology, Stanford University School of Medicine, 875 Blake Wilbur Dr Clinic D, Stanford, CA MC 584794305 USA; 2 Gladstone Institutes, San Francisco, CA USA

**Keywords:** Salivary glands, Salivary gland stem/progenitor cells (SSPCs), Clinical implications, Regenerative medicine

## Abstract

Salivary gland stem/progenitor cells (SSPCs) hold significant potential for regenerative medicine, especially for patients suffering from salivary gland dysfunction due to various causes such as radiation therapy, Sjögren’s syndrome, and aging. This review provides a comprehensive overview of SSPCs, including their characteristics, isolation, culture techniques, differentiation pathways, and their role in tissue regeneration. Additionally, we highlight recent advances in cell- and tissue-based therapies, such as SSPC transplantation and bioengineered organ replacements. The challenges in translating SSPC research into effective clinical therapies are also discussed, alongside proposed solutions and future research directions.

## Background

The salivary glands (SGs) play a crucial role in maintaining oral health by producing saliva, which is essential for digestion, lubrication, and antimicrobial defense. Both humans and mice have three pairs of major SGs (parotid, submandibular, and sublingual glands) and several minor SGs (Chason and Downs 2024; Ghannam and Singh 2024). Each major SG has unique anatomical and functional properties that contribute differently to saliva production and secretion. The glands are composed of functional units called acini, which are clusters of specialized epithelial cells known as acinar cells. Acinar cells are responsible for the production of saliva precursors, which are then modified and transported through a network of ducts lined with various types of epithelial cells.

Dysfunction of salivary glands due to conditions such as Sjögren’s syndrome, radiation therapy for head and neck cancers, or congenital defects can lead to significant morbidity, including xerostomia, difficulties in swallowing and speaking, increased dental caries, and oral infections. There is evidence showing the presence of salivary gland stem/progenitor cells (SSPCs) in the adult salivary gland that has promising potential for repairing or regenerating damaged salivary gland tissue (Emmerson and Knox 2018; Aure et al. 2015). Understanding the characteristics of SSPCs is fundamental to advancing regenerative medicine approaches, thereby restoring normal salivary gland function and improving patients’ quality of life.

In this review, we mainly focused on the mammalian SSPCs. We first describe the key characteristics of SSPCs and then discuss the latest findings on SSPCs isolation and culture methods, differentiation regulation, tissue regeneration capabilities, and clinical applications. By providing a comprehensive overview of the current state of SSPC research, we aim to highlight the potential of these cells in regenerative therapies for salivary gland dysfunction.

## SSPCs in salivary gland development: from gestation to adulthood in mammals

The development of the salivary gland is a complex and tightly regulated process that begins early in gestation and continues during adulthood. The major SGs undergo several stages of development involving cellular differentiation, tissue morphogenesis, and functional maturation. Although SGs share a common mechanism of branching morphogenesis during embryonic development, adult SGs exhibit distinct histological structures and specialized functions (Denny et al. 1997).

## Embryonic development

**Fig. 1:**
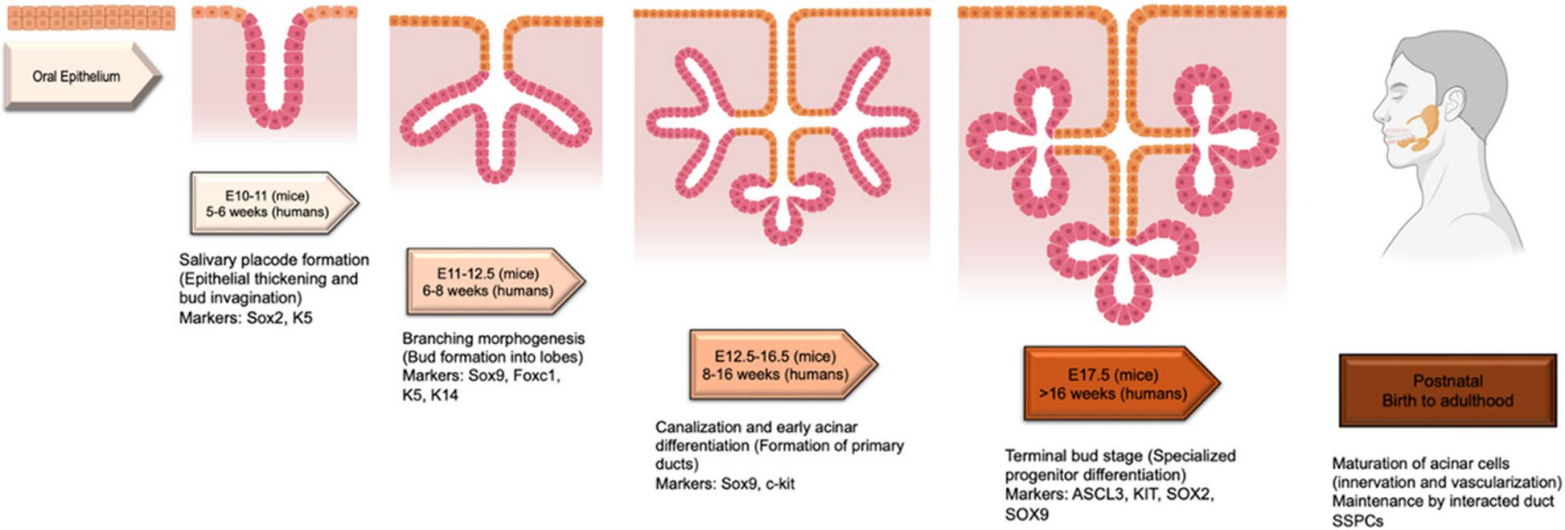
The figure above illustrates the developmental stages of salivary glands in mice and humans, highlighting the corresponding processes and SSPC markers at each stage. This figure was created with Biorender.com

In mice, SG development begins on embryonic days (E) 9~10, whereas in humans, it starts at 5~6 weeks of gestation (Suzuki et al. 2021). During this initial stage, the oral epithelium thickens to form a salivary placode, which invaginate into the underlying mesenchyme to create bud (Patel et al. 2006; Tucker 2007). This process is regulated by signaling pathways such as FGF (Fibroblast Growth Factor), SHH (Sonic Hedgehog), and Wnt signaling (Liu and Wang 2014). By E11-E12 in mice and 6~8 weeks in humans, multipotent progenitor cells expressing markers like Sox2 and K5 emerge within the oral epithelium and respond to these molecular cues to initiate gland development (Patel et al. 2006) (Fig. 1).

As epithelial branching morphogenesis progresses (E12.5-E16.5 in mice; 8~16 weeks in humans), the bud undergoes proliferation and branching to form the primary ducts and early lobes of the gland. Key markers such as Sox9, Foxc1, c-kit, and K14 characterize emerging progenitor populations (Aure et al. 2015; Lombaert et al. 2008). These cells contribute to the formation of glandular structures, with c-Kit + cells in the end buds giving rise to proximal (ductal) and distal (acinar) lineages. During this period, epithelial-mesenchymal interactions mediated by FGF10 and ECM proteins refine the glandular architecture (Patel et al. 2006; Jaskoll et al. 2004) (Fig. 1).

## Terminal bud and postnatal development

By the terminal bud stage (E17.5 onwards in mice and after 16 weeks in humans), progenitor populations become more lineage-restricted. Markers such as ASCL3 + , KIT + , SOX2 + , and SOX9 + characterize progenitors that differentiate into the functional units of the gland. Acinar cells, which produce saliva, and ductal cells, which facilitate its transport, arise from these specialized progenitors (Chason and Downs 2024; Knox et al. 2010) (Fig. 1).

Postnatally, the major SGs continue to mature. From birth to 4 weeks in mice and birth to 2~3 years in humans, the glands undergo further differentiation, vascularization, and innervation, which are essential for glandular function (Emmerson and Knox 2018). The composition of saliva evolves during infancy, increasing in specific enzymes and proteins necessary for digestion and oral health (Tucker 2007) (Fig. 1).

## Role of SSPCs in homeostasis and repair

In mature glands, progenitor-like cells residing in the intercalated ducts retain regenerative potential. These cells can differentiate into both acinar and ductal cells during homeostasis and repair. For instance, Sox2 + and c-kit + progenitors contribute to tissue maintenance, while Sox9 + progenitors play critical roles during regeneration after injury (Lombaert et al. 2008; Emmerson et al. 2018). Studies have demonstrated that these cells, supported by niche signals such as FGF and TGF-β are crucial for long-term glandular function and repair (Aure et al. 2015; Jaskoll et al. 2004).

## Comparative insights across species

Salivary glands are vital not only in mammals but also in other vertebrates, with various adaptations and functions across different species. For instance, fish have mucous-secreting cells that act as salivary glands to provide lubrication and protection. Amphibians have merged tongue and salivary glands with lingual cells containing secretory granules. It provides dual functions: catching prey and moistening food, which is vital for aquatic and terrestrial living. Reptiles use modified salivary glands to produce venom and for lubrication. Birds use their salivary glands to build nests and food lubrication. Altogether, salivary glands have evolved over time, adapting to the requirements of the various species; however, the presence and role of SSPCs in the salivary glands of these vertebrates have not yet been elucidated. Investigating these evolutionary parallels could further elucidate the molecular drivers of salivary gland formation and SSPCs function.

## Adult salivary gland stem/progenitor cells: Identification methods and key markers

**Fig. 2:**
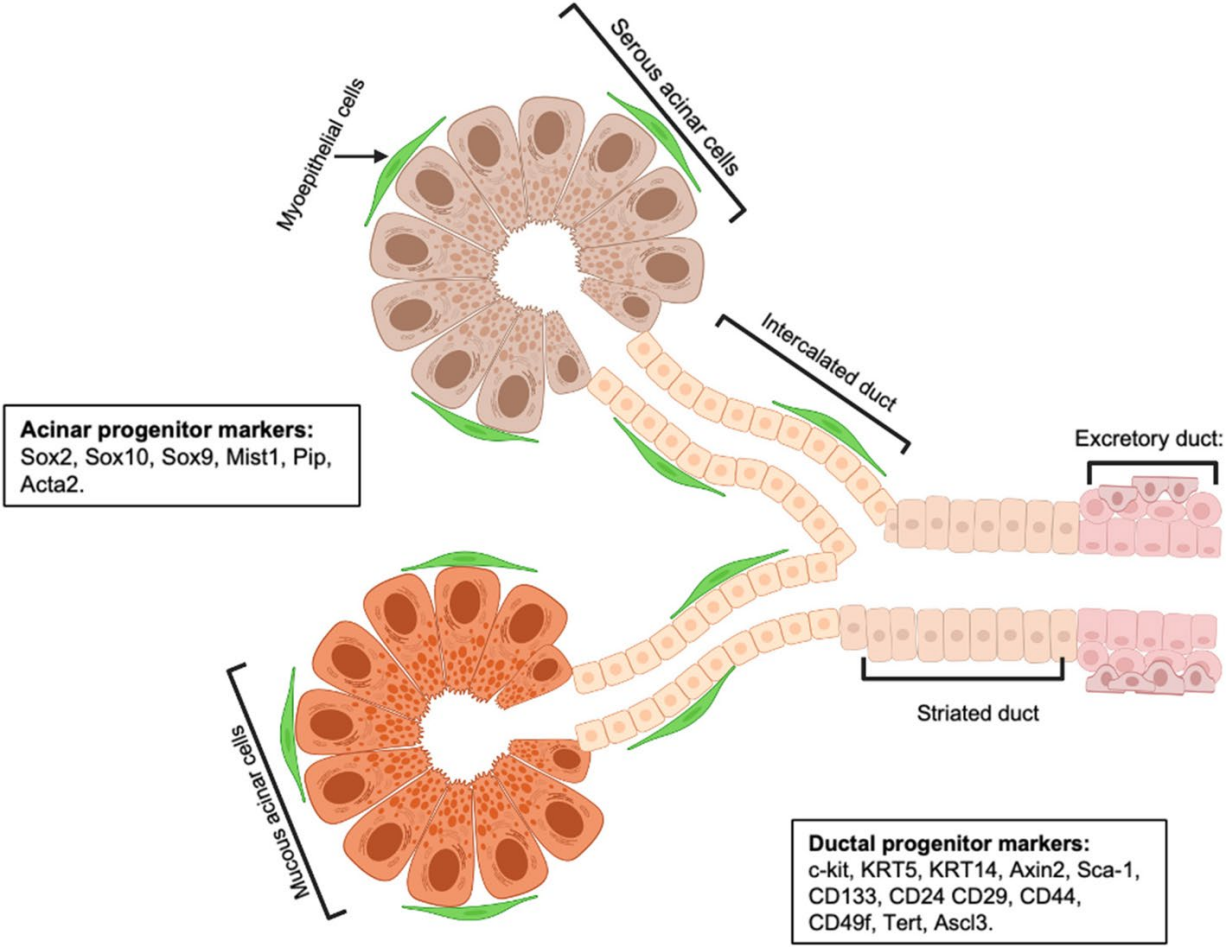
Schematic of generic adult salivary gland structure and compartments showing acinar and ductal adult SSPCs markers. This figure was created with Biorender.com

Adult SSPCs are a specialized population of cells that contribute to the maintenance, repair, and regeneration of salivary glands (SGs) in response to injury or during homeostasis. These cells are found primarily in the intercalated ducts and other niche environments within the glands (Fig. 2). Here, we discuss several methods of identification for adult SSPCs.

The characteristics of well-studied hematopoietic stem cells (HSC), such as their rarity, quiescent state, and ability to asymmetrically divide, serve as a template to identify stem cells of other tissues (Beumer and Clevers 2024; Nunes and Loeffler 2024). From the HSC point of view, adult stem cells are slow-cycling cells in a homeostatic condition, limiting their division to avoid DNA replication errors (Beumer and Clevers 2024). Based on this, several attempts have been made to identify SSPCs using ^3^H-thymidine, bromodeoxyuridine, and histone-green fluorescent protein fusion protein for an extended period to identify slow-cycling cells (Denny and Denny 1999; Denny et al. 1993; Man et al. 2001; Kwak and Ghazizadeh 2015; Pringle et al. 2013). These studies supported that during homeostasis, intercalated ducts differentiate into acinar and granulated ductal cells, and therefore, intercalated ductal cells can be considered as transient amplifying progenitor cells (Denny et al. 1993; Man et al. 2001; Yagil et al. 1985). A study by Chibly et al. also revealed the presence of putative salivary gland progenitor cells using labeled retaining cells (LRC) (Chibly et al. 2014). However, these studies could not distinguish between potential quiescent stem/progenitor cells and other cells that are either slow-cycling during the experiment or have differentiated without further division in homeostatic conditions. Therefore, it is insufficient to conclude that LRCs are SSPCs. Salivary glands are slow-turnover tissues that increase proliferation in response to damage to replace the lost cells and then return to their low-level maintenance when homeostasis is restored. Therefore, LRC assays are not the best way to identify salivary gland stem cells.

Another method, genetic lineage tracing, is a powerful tool for understanding salivary gland stem/progenitor cells. Lineage tracing involves genetically labeling putative progenitors and their descendants using a fluorescent protein or LacZ reporter. These experiments rely on the specificity and accuracy of cell-specific promoters attached to the Cre recombinase, which permanently marks the target cells and their progeny, even if they cease to express the specific marker (Kretzschmar and Watt 2012). This method helps to elucidate lineage relationships, assess long-term self-renewal potential, and monitor cell behavior in vivo. The timing of Cre recombinase activation and the specific marker’s spatial and temporal expression pattern are critical factors for interpreting lineage contributions. Since Cre activation is a permanent and heritable event, results must take into consideration potential changes in gene expression over time. Cre may be expressed in one set of cells at a particular time and in different cells at another, complicating the interpretation of lineage relationships. An inducible Cre system can offer temporal and spatial control by selectively inducing Cre in adult tissues via active promoters during embryonic development to address this challenge.

Using genetic lineage tracing, many groups have shown that salivary gland ductal progenitor cells (KRT14 + , c-kit + , Axin2 + , KRT5 +) contribute to the formation and maintenance of glandular ducts throughout life (May et al. 2018; Kwak et al. 2016; Weng et al. 2018) while other studies suggest that acinar cells Mist1 + (Weng et al. 2018), Sox2 + (Aure et al. 2015; Emmerson et al. 2018), Pip + (Maruyama et al. 2016), Acta2 (May et al. 2018; Song et al. 2018) self-duplicate during adult salivary gland homeostasis. Additional putative acinar progenitor cell markers include Sox9 + (Xu et al. 2022), Sox10 + (Athwal et al. 2019), and additional putative ductal progenitor markers include Sca1 + (Lombaert et al. 2008; Purwanti et al. 2011), CD133 + (Nanduri et al. 2011) CD44 + (Banh et al. 2011), CD29 + (Nanduri et al. 2011; Nanduri et al. 2014; David et al. 2008; Sato et al. 2007) CD24 + (Nanduri et al. 2011; Nanduri et al. 2013; Xiao et al. 2014) CD49f + (Nanduri et al. 2011; Nanduri et al. 2014; David et al. 2008; Nanduri et al. 2013; Sato et al. 2007; Okumura 2003) (Fig. 2).

By using ASCL3^EGFP−Cre^ mouse model, Rugel-Stahl et al. demonstrated that ASCL3 + progenitor cells are presented in the salivary gland, and these ASCL3-expressing cells can generate both duct and acinar cell descendants (Rugel-Stahl et al. 2012). Additionally, studies from our lab, using a mouse strain expressing regulated Cre^ERT2^ recombinase from the endogenous Tert locus, Guan et al. identified a distinct telomerase-expressing (Tert^High^) cell population located in the ductal region of the adult submandibular gland (SMG). These Tert^High^ cells contribute to ductal cell renewal during SMG homeostasis and to both ductal and acinar cell regeneration after radiotherapy (Guan et al. 2024) (Fig. 2).

## Isolation and culture of salivary gland stem/progenitor cells (SSPCs)

There are various methods for isolating SSPCs, each with its own advantages and challenges. Here are some of the commonly used methods for isolating SSPCs:

1. **Tissue mincing and enzymatic digestion:** This method involves collecting salivary gland tissues and mincing them into small pieces. The minced tissue is then subjected to enzymatic digestion using enzymes like collagenase, trypsin, dispase, or a Miltenyi tissue digestion kit. The resulting cell suspension is filtered, centrifuged, and then passed through a filter to obtain single-cell suspension. However, this method may result in a heterogeneous population of cells, including SSPCs, and can sometimes damage these cells or result in a low yield of viable cells (Beucler and Miller 2019; Yoon et al. 2022).
2. **Fluorescence-activated cell sorting (FACS)**: This method can purify a pure population of SSPCs by labeling the isolated cells with fluorescently tagged antibodies against specific cell surface markers known to be expressed by these cells (e.g., c-Kit, KRT14, and KRT5) and then sorting them using a flow cytometer based on the fluorescence intensity. This results in a highly pure population but requires access to sophisticated equipment and a prior knowledge of specific stem cell markers (Sutermaster and Darling 2019).
3. Side population sorting is another method to isolate stem cell populations using FACs. This method uses a DNA-binding dye such as Hoechst 33,342 to stain the isolated heterogeneous population and then sort the side population cells. However, this method is technically challenging, and interpreting side population data requires careful control (Golebiewska et al. 2011).
4. **Magnetic-activated cell sorting (MACS)**: This method purifies a pure population of SSPCs by labeling the cells with magnetic beads conjugated to antibodies against stem cell-specific markers and then passing them through a magnetic column. MACS is less expensive and easier to perform than FACS, but the purity of cells may be lower (Sutermaster and Darling 2019).
5. Other methods include using genetically modified animals expressing a fluorescent protein or a reporter gene under the control of a stem cell-specific promoter. However, this method requires the generation of transgenic animals, which is more complex and expensive than other methods (Furukawa et al. 2015).

Each method has its specific applications depending on the research goals, available equipment, and the specific type of stem cells being targeted. Often, a combination of these methods is used to achieve optimal results.

## Salivary gland organoid culture

Organoids are advanced, tissue-engineered in vitro models that closely replicate the structural complexity and functional characteristics of their corresponding in vivo tissues. These cell-derived systems provide a physiologically relevant platform for studying tissue-specific processes, offering a significant potential for modeling human development, disease pathogenesis, and therapeutic response under controlled laboratory conditions. Salivary gland organoids can be cultured from SSPCs. Several methods are used for organoid culture. The details of different methods are well described in the review by Zhao et al. (2021), which addresses critical factors for producing robust organoids including considerations related to cell isolation and seeding, the choice of matrix and soluble factors, as well as the influence of physical cues, and integrated culture systems. Some of the latest developments in salivary organoid culture include studies by Zhao et al. (2021), Kim et al. (2021), Yoon et al. (2022), and Tanaka et al. (2022).

## Recent advances in salivary gland regeneration for clinical applications

There is growing interest in utilizing stem cell therapies to restore salivary gland function following radiation damage. Current clinical trials focus on mesenchymal stem cells (MSCs) (NCT04776538) and the patient's own salivary stem cells, though less attention has been directed toward salivary gland progenitor cells. While still in the early stages, clinical applications of stem cells for salivary gland regeneration are gaining traction, with several pilot studies and trials showing encouraging results. For instance, Lynggaard et al. demonstrated that mesenchymal stem/stromal cell therapy could reduce radiation-induced xerostomia (Lynggaard et al. 2022). Their study on the long-term safety of autologous MSCs in treating radiation-induced xerostomia through the MESRIX Phase I/II trial suggests it has significant potential. Furthermore, autologous adipose tissue-derived MSC (ASC) therapy has been shown to be both safe and effective, with a clinically relevant impact on xerostomia-related symptoms (Marinkovic et al. 2023; Jakobsen et al. 2024). These studies aim to assess the safety, feasibility, and efficacy of stem cell-based therapies in restoring salivary gland function for patients with gland hypofunction. The early findings offer hope that stem cell therapy could eventually be a viable treatment option for managing radiation-induced salivary gland dysfunction.

c-Kit is considered a potential marker for isolating salivary gland stem cells, though its reliability remains under debate. Some studies, such as those by Histomi and Kwak, reported that c-Kit + cells, which appear in the salivary glands after duct ligation, exhibit multipotency and can differentiate into salivary gland cells (Okumura 2003; Hisatomi et al. 2004; Kwak et al. 2018). However, long-term in vivo lineage tracing has revealed that while c-Kit marks ductal cell lineages, it does not mark acinar cells, suggesting that c-Kit is more likely a marker for progenitor cells rather than stem cells. Despite this, c-Kit-positive cells have demonstrated the ability to acquire stem cell-like multipotent characteristics during tissue regeneration following injury, although the mechanisms driving this are still unclear (Xiao et al. 2014; Ninche et al. 2020; Rocchi et al. 2021).

Research from our lab and others has identified the existence of salivary stem/progenitor cells (SSPCs) in the submandibular gland (SMG) of adult mice, which can restore salivary function when transplanted into irradiated SMGs of recipient mice (Lombaert et al. 2008; Xiao et al. 2014). However, the rarity of adult SSPCs presents a challenge in their therapeutic application. To address this, we have systematically characterized the genes that are differentially upregulated in SSPCs, uncovering key targets for therapeutic exploitation (Xiao et al. 2014). Our group discovered that the enzyme ALDH3A1 is highly expressed in SSPCs (Banh et al. 2011; Xiao et al. 2013). By administering Alda-89, an ALDH3A1-specific small-molecule activator, we were able to increase the number of SSPCs, preserve acinar structures, and restore salivary function in irradiated mice (Xiao et al. 2013). Notably, Alda-89 infusion did not result in head and neck cancer protection from radiation (Xiao et al. 2013), indicating the safety and potential of ALDH3A1 activation as a novel approach for preventing and treating RT-induced xerostomia.

While these advances offer great promise, several challenges remain. These include optimizing stem cell delivery methods, ensuring the long-term viability and integration of transplanted cells, and mitigating risks such as tumorigenesis. Continued research is necessary to address these obstacles, but the initial results indicate that stem cell therapy could provide a promising solution for salivary gland disorders in the near future.

## Limitations and future directions of SSPCs research

A significant limitation in SSPC research lies in the ambiguity surrounding key markers, such as c-Kit, which has shown conflicting roles in identifying true stem/progenitor cells versus transiently amplifying progenitor. This inconsistency underscores the need for refined characterization and validation of SSPC-specific markers to ensure accuracy and reproducibility across studies. Additionally, the majority of SSPC studies have been conducted in murine models, which, while valuable for foundational insights, present limitations in directly translating findings to human biology and clinical applications. Bridging this gap requires more robust investigations into human SSPCs to better understand their behavior, markers, and therapeutic potential.

Future research directions in SSPC studies should focus on leveraging advanced technologies to overcome existing challenges and broaden therapeutic potential. Single-cell transcriptomics can play a pivotal role in resolving SSPC heterogeneity, providing deeper insights into the molecular profiles of distinct SSPC populations, and identifying novel therapeutic targets. Longitudinal lineage tracing is essential to track SSPC dynamics under conditions of homeostasis, injury, and repair, enabling a better understanding of their regenerative behavior and lineage relationships over time. Additionally, developing scalable organoid systems is critical for advancing bioengineered salivary gland replacements, facilitating large-scale therapeutic applications, and modeling disease processes with greater physiological relevance. These approaches collectively hold promise for translating SSPC research into effective clinical therapies.

## Conclusions

Salivary gland stem/progenitor cells hold immense promise for revolutionizing the treatment of salivary gland dysfunctions and improving oral health for millions. While significant challenges remain in definitively identifying, characterizing, and translating basic research findings into clinical applications, the field is experiencing rapid progress. Continued research efforts hold the potential to unlock the therapeutic power of SSPCs, offering hope for patients suffering from salivary gland disorders.

## Data Availability

Not applicable.

## Abbreviations

SSPCs: Salivary gland stem/progenitors’ cells
SG: Salivary gland
FGF: Fibroblast growth factor
SHH: Sonic Hedgehog
EGF: Epidermal growth factor
DNA: Deoxyribonucleic acid
ASCL3: Achaete-Scute Family BHLH Transcription Factor 3
LRC: Label retaining cells
KRT: Keratin
SMG: Sub-mandibular gland
FACS: Fluorescence-activated cell sorting
MACS: Magnetic activated cell sorting1
MSCs: Mesenchymal stem cells
ASC: Adipose tissue-derived stem cell therapy
ALDH3A1: Aldehyde dehydrogenase 3 family member A1
